# Retinoic acid promotes the endogenous repair of lung stem/progenitor cells in combined with simvastatin after acute lung injury: a stereological analysis

**DOI:** 10.1186/s12931-015-0300-9

**Published:** 2015-11-11

**Authors:** Ce Yang, Xuetao Yang, Juan Du, Haiyan Wang, Haisheng Li, Ling Zeng, Wei Gu, Jianxin Jiang

**Affiliations:** State Key Laboratory of Trauma, Burns and Combined Injury, Institute of Surgery Research, Daping Hospital, Third Military Medical University, Chongqing, 400042 China; Research Institute of Surgery, Daping Hospital, Third Military Medical University, Changjiang Zhilu, Daping, Chongqing, 400042 China

**Keywords:** stem/progenitor cells, retinoic acid, simvastatin, apoptosis, regeneration, trauma and injury

## Abstract

**Background:**

The treatment of acute respiratory distress syndrome (ARDS), most commonly seen during the organ dysfunction remains unsatisfied. Presently, the stem/progenitor cell-based endogenous repair has been aroused attention enormously. This report investigated the effects of retinoic acid (RA) plus simvastatin (SS) with respect to dynamics of lung repair cells as well as to elucidate the underlying mechanism.

**Materials and methods:**

The experimental Sprague–Dawley rats were divided randomly into normal control (control), sham operated (sham), ARDS, ARDS + vehicle and ARDS + RA + SS groups. ARDS was reproduced through hemorrhagic shock/resuscitation (shock) and subsequent intratracheal LPS (4.5 mg/kg, Escherichia coli serotype O55: B5) injection. The rats were treated by intragastric administration of RA (2 mg/kg/day) and SS (2 mg/kg/day) for 5 days in the ARDS + RA + SS group. Seven days after the first RA-SS injection, a right lower lobe of lung was sampled for histological analysis concerning systemic uniform random sampling method. Immunohistochemistry of inflation-fixed lungs for alveolar type 1 (AT1), alveolar type 2 (AT2) and Clara cells was measured by AQP5, Pro-SPC and CCSP staining respectively. The alveolar cell proliferation and apoptosis were analyzed with Ki67 staining and terminal deoxylnucleotidyl transferase mediated-dUTP nick end labeling (TUNEL) method. Meanwhile, the alveolar cell numerical and surface density (alveolar cells, AT1, AT2, Clara, proliferating and apoptotic cells) were evaluated by stereology.

**Results:**

RA-SS compound exerted anti-inflammatory and pro-repairing effects on respiratory tracts in ARDS induced by hemorrhagic-endotoxin shock. The numerical density and surface density of alveolar cells, AT1 cell fraction, and numerical density of AT2 and Clara cells were significantly increased after treatment with RA-SS compound in ARDS. Concurrently, the Ki67+ alveolar cells were obviously increased while the TUNEL+ alveolar cells were reduced, which was correlated with the attenuation of inflammatory injury and functional repair in injured lung tissues.

**Conclusions:**

Our data convincingly indicated that the prophylactic and therapeutic treatment of RA plus SS had obvious beneficial effect on the remodeling/regeneration of injured pulmonary tissues, suggesting that the underlying mechanisms are related to the re-balance between regeneration and apoptosis in lung stem/progenitor cells.

## Background

Acute respiratory distress syndrome (ARDS) remains a severe disease threatening millions of traumatic infectious patients in intensive care units, accounting for 60, 0000–70, 0000 patients and 40 % - 70 % of mortality among this cohort [1]. The efficient diagnostic and therapeutic capabilities in ARDS are of great importance for the treatment in multiple organ dysfunctions. Hastening the repair process of damaged pulmonary tissues has been thought to be the most effective strategy for ARDS. However, compelling evidence indicates that the remedy of ARDS based on the ventilation function support and anti-inflammatory treatment remains unsatisfied [2–5]. Very recently, the remodeling of functional pulmonary repair for the treatment of ARDS has been paid much attention within the recovery of gas exchange. The potential measures to realize the repair courses of injured adult lung tissues are to activate the self repairing potential, and improve the local pulmonary microenvironment so as to promote the reconstruction of breathing function [6, 7]. During these complicated courses, the principal biological event is that lung stem/progenitor cells are synergistically involved in the repair of injured lung tissues.

Lung stem/progenitor cells, distributed in the predetermined microenvironment named niche [8, 9], may be the principal source of local repair cells in the adult respiratory tissues in rodents and humans. Although there remains lack of the specific molecular markers for lung stem/progenitor cells, their isolation and culture seems difficult, and their classifications are also controversial [10], they have been widely approved for maintaining of pulmonary structural stability and functional repair. Desai et al., has clearly presented the differential roles of alveolar stem/progenitor cells in lung development and renewal [10], supplying the valuable indication for their role in the remodeling and regeneration in ARDS.

Previous studies have demonstrated that retinoic acid (RA) is involved in the lung development, especially the alveolar genesis via morphological branches of fetal lungs and developmental gene-related alveolar separation [11, 12], which is further confirmed by the RA receptor knockout mice. All-trans retinoic acid (ATRA) is therapeutically effective by inducing alveolar regeneration in rodent animals in a species- and dose-dependant manner [13–15], suggesting its potential repairing capacity. Meanwhile, simvastatin (SS), an inhibitor of for 3-hydroxy-3-methylglutaryl coenzyme A (HMG-CoA) reductase, plays an active pharmacological efficacy in anti-inflammation and recovery of epithelial function in repair processes of injured pulmonary tissues [16, 17]. Simvastatin given intravenously could increase the amount of proliferating alveolar epithelial cells [16, 18], as well as the promotion of pulmonary tissue restoration via exogenous epithelial progenitor cells in a synergistic way in injured lungs [19]. The demonstration that SS exerts benefit in the endotoxin-injured human lung is also persuasive [17]. Therefore, the synergistic treatment of RA and SS maybe benefits for the homeostasis of pulmonary microenvironment and remodeling of injured lung tissues in the modulation of pulmonary stem/progenitor cells.

**Fig. 1:**
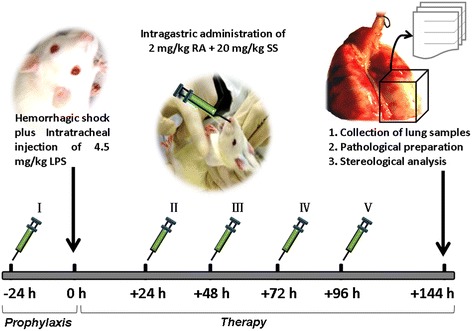
Schematic illustration of the experimental procedure of retinoic acid (RA) and simvastatin (SS) compound and stereological analysis for acute respiratory distress syndrome (ARDS) in rats

Thus, we hypothesized that the compound of RA plus SS (RA-SS) improves the injured pulmonary microenvironment and dynamics of lung stem/progenitor cells in the functional repair of respiratory tract. Therefore, we choose a hemorrhagic shock/resuscitation plus LPS challenge model to observe the effects of RA-SS on the number of alveolar, AT1, ATII, Clara, proliferating and apoptotic cells in a stereological method, and their relationship with the repair of injured lung tissues (Fig. 1). These data provided new insights into the protective effect of RA-SS in the re-balance between proliferation and apoptosis of lung stem/progenitor cells as well as its effective prophylaxis and therapy in ARDS.

## Materials and methods

### Ethical approval

All animal procedures were approved by the Institutional Animal Care and Use Committee of the Third Military Medical University (Permit No. SCXK (yu) 2007017) and in accordance with the recommendations in the ARRIVE Guidelines for the care and use of experimental animals. We have made great efforts to minimize the number of animals used and their suffering.

### Experimental animals and grouping

Adult Sprague–Dawley (SD) rats (6–8 weeks old, 250–300 g, n = 32) were used in this study. The animals were housed in hanging wire mesh cages in the accredited animal facilities of the Research Institute of Surgery. The animal room was specific pathogen-free and was maintained with controlled temperature, humidity and lighting (12-hour light–dark cycles). All animals were given rat chow and tap water ad libitum. The animals were divided into normal control (normal), sham operated (sham), ARDS (hemorrhagic shock/resuscitation + LPS), ARDS + vehicle and ARDS + RA + SS groups. Six rats were used for each time point. Animals sacrificed before the time points were replaced by other corresponding SD rats.

### Acute respiratory distress syndrome (ARDS)

ARDS was reproduced through hemorrhagic shock/resuscitation (HR) in combination with intratracheal administration of bacterial LPS (*Escherichia coli* serotype O55: B5, Difco Laboratories, Detroit, MI) as previously described [20, 21]. Briefly, after anesthetization with intraperitoneal pentobarbital sodium (60 mg/kg), rats were fixed on the supine position. At the upper one third sites of femoribus internus, dissection was carried out through an oblique incision to expose femoral artery. After careful blunt dissection, the distal site of femoral artery was ligated while a small incision of its proximal site was made to establish a three-way silica gel pipe followed with the slow injection of heparin solution (1000 U/kg). Five minutes later, the three-way pipe was connected with hemomanometer for monitoring of animal blood pressure (BP). Then, blood was slowly drawn with a syringe to make the BP descend at the level of 40 mmHg within 10 min. The status of 35 - 40 mmHg was kept for 90 min, followed with slow transfusion with previous losing blood and equal volume of ringer solution within 15 min. Finally, with the aid of trachea cannula, about 150 micro liter LPS (4.5 mg/kg) was given immediately. Finally, all the cannulas were pulled out and the incision was sutured with sterilization. The sham-operated animals underwent surgical procedures and intratracheal injection of equal volume of sterilized physiological saline without hemorrhagic shock/resuscitation and LPS challenge.

### Treatment with RA and SS

The lyophilized powder of RA from Sigma is freshly dissolved in olive oil, while SS (Merck Sharp & Dohme, MSD, Hangzhou, China) is dissolved in sterilized saline. Animals were treated with RA-SS by intragastric administration. The rats in the ARDS + RA + SS group were treated with RA at 2 mg/kg/day and SS solution at 20 mg/kg/day for five days before and after ARDS, while those in the ARDS + vehicle group were treated with equal volume of olive oil and sterilized saline at the same time. The first treatment began at 24 hrs before ARDS. The treatment time is between 8:00 a.m. and 11:00 a.m.

### The body weight

The body weights were recorded within the whole experiment. The body weight changes were calculated according to the following equation: The percent of body change = (body weight after ARDS - body weight before ARDS) / body weight before ARDS %.

### Collection of samples

The rats were anesthetized by intraperitoneal injection of pentobarbital sodium (60 mg/kg) and kept on a surgical board at the given time points. Concerning the quantitative analysis of lung structure in stereological analysis, a complete fixation is a prerequisite to avoid bias in tissue dimensions and structural details. We selected high-quality lung fixation by a combined perfusion via closed loop and trachea. First, the pulmonary circulation was flushed with 40 ml cold (4 °C) heparinized phosphate-buffered saline (PBS) via right ventricle injection to remove blood elements. The right ventricle was cut open for bleeding. After the lung was bleached, fifty milliliter of 4 % paraformaldehyde solution was then used for pre-fixation. Meanwhile, the trachea was inflated with 3–5 ml air occasionally to guarantee the vessel expansion during the lavage courses. Second, the trachea then began to fill with 4 % paraformaldehyde solution until the hydrostatic pressure reached 25 cm water column. Finally, the lungs filled with paraformaldehyde solution were ligated at the upper site of trachea, and immersed in 4 % paraformaldehyde solution at 4 °C for 48 h.

Then, the right lower lobe of the lung was snapped. According the stereological criteria of uniform isotropic random (UIR) [22, 23], the lobe was incised 9–11 tissue blocks for histological analysis, whose position within the whole and orientation are both random. The samples were progressively dehydrated with ethanol and dimethylbenzene and then embedded with paraffin. Lung tissues were prepared for paraffin sections and hematoxylin and eosin (HE) or immunohistochemistry staining. All histopathological analyses for each group were performed in a blinded fashion.

### Histological examination

The animals were sacrificed to preserve lung architecture at corresponding time points. Lung tissues fixed in 4 % paraformaldehyde solution were embedded in paraffin, and cut into 2 μm thick sections. Sections were stained with HE. The degrees of lung injury were evaluated blindly by a pathologist under a light microscope, according to the following criteria: sepals thickening, alveolar and interstitial edema, hyaline membranes, inflammatory cell infiltration, and small airway epithelial injury.

### Determination of AQP5, Pro-SPC and CCSP, Ki67 after ARDS

Expression of AQP5, Pro-SPC CCSP and Ki67 was determined by a standard immunohistochemistry assay [24]. Briefly, sections were given antigen restoration with high-pressure steam for 5 min, washed in PBS containing 0.1 % BSA and permeabilized with PBS-blocking buffer (PBS with 0.1 % BSA and 0.3 % Triton X-100) for 10 min at room temperature. Then sections were rinsed with 0.01 mol/L PBS for three times. After blocked with 10 % normal donkey serum for 30 min at 37 °C, the tissues were incubated with anti-AQP5 (1: 500, Abcam, #ab78486, Newcastle, UK), Pro-SPC (1:2000, Millipore, #AB3786, USA), CCSP (1:1000, Millipore, #07-623, USA) or Ki67 (1:200, Abcam, #ab66155, Newcastle, UK) antibodies diluted in 0.01 mol / L PBS at 4 °C overnight. After washing five times with an excess of PBS, The primary antibodies were detected with AF488-conjugated donkey anti-rabbit immunoglobulin (1: 400; #A21206, Invitrogen, USA), or AF594-conjugated donkey anti-goat immunoglobulin (1: 400; #A11058, Invitrogen, USA). Finally, the tissues were further stained with 2-(4-amidinophenyl)-6-indolecarbamidine (DAPI) dihydrochloride (1:10000, #C1002, Beyotime, Jiangsu, China) for stereological analysis.

### In situ cell death detection analysis of apoptosis by TUNEL

To detect DNA fragmentation in cell nuclei, TUNEL reaction was applied to the paraffin sections by using a Kit (Roche, Germany). After deparaffinization, the sections were treated with 0.1 % Triton X-100 and 0.1 % sodium citrate for 8 min on ice. After treatment with 0.3 % H_2_O_2_ in methanol for 10 min, the sections were incubated with TUNEL reaction mixture for 60 min at 37 °C. Further incubation with horseradish peroxidase (POD, 1:500) was performed for 30 min at 37 °C. The apoptotic cell evaluation was performed with a modified technique previously described [25].

### Stereological analysis of lung tissue and cells

Concerning the criteria of stereological parameters and study designs in the respiratory tract [26], the numerical density and surface density of alveolar cells and liner intercept (Lm) were determined on the sections with HE staining, while surface fraction of AT1 cells, numerical density of AT2, Clara, proliferating and apoptotic cells were given on the sections with immunohistochemistry or TUNEL staining.

### Alveolar cell numerical density

Under the microscope with high magnification, the target fields of vision are located in the area of alveolar tissues with an interval of 1.5 mm both in horizontal and vertical axes. The selected visions are in accordance with the left-right and up-down snake-like sequence. Secondly, in the adobe Photoshop 7.0.1 procedure, the selected tissue pictures were overlaid with unbiased counting frames and cross line segments. The number of adopted intersections is counted as Q. The fraction of alveolar surface area = Q/144. the alveolar surface area (S) = area of unbiased counting frame (0.017 mm^2^) × Q/144. Then, the number of adopted alveolar cells was counted as N_AC_. All the adopted cells are in the counting frames except those intersected with the forbidden lines. The numerical density of alveolar cells = N_AC_/S.

### Alveolar cell surface density

Further, the selected tissue pictures were overlaid with unbiased counting frames and line segments. The area of unbiased counting frame, whose actual area is 0.017 mm^2^, covers 50 % of the total area in a tissue picture. The test macro includes 42 line segments and 84 points. A line segment with 23 μm in length includes midline, left and right endpoints, with blocking lines residing in the endpoints and extension lines on the two sides. The number of intersections between the line segments and the alveolar walls was counted as I. The total length of line segments in the counting frame was calculated as L1. Finally, alveolar cells surface density = 2I/L1.

### Alveolar Lm

To evaluate the status of alveolar ectasia, we further counted the right endpoints of the line segments falling in the alveolar spaces in the counting frame as N_RE_. The total length of line segments falling in the lung tissues in the counting frame was calculated as L2. Lm = 2 L2 × N_RE_/I.

### AT1 cell surface fraction

Similar to the analysis of alveolar cell surface density, the selected tissue pictures were overlaid with unbiased counting frames and line segments. The number of intersections between the midline and the interalveolar septum covered with AT1 cells was counted as P1, while that of intersections between the midline and the interalveolar septum denuded or uncovered with AT1 was counted as P0. The criteria of adopted intersections include the complete interception points as well as the incomplete interception points only residing in the upper- and right-sides. The number of target visions in each animal at least reach 30. The surface fraction of AT1 cells = P1/(P_0_ + P_1_).

### AT2 cell numerical density

Similar to the stereological analysis of AT1 cells, the selected tissue pictures were overlaid with unbiased counting frames and cross line segments. The total number of test points is 144. The adopted intersections should both cover the point of intersection and right superior quadrant. The number of adopted intersections is counted as Q. The fraction of alveolar surface area = Q/144. the alveolar surface area (S) = area of unbiased counting frame (0.017 mm^2^) × Q/144. Then, the number of adopted AT2 cells was counted as N_AT2_. The adopted AT2 cells should express pro-SPC in the cytoplasm with a DAPI+ nucleus. The shape of positive granules in the photos should be round, semi round or elliptical. The round- or cube-shaped cells reside in the alveolar wall. All the target cells are in the counting frames except those intersected with the forbidden lines. The numerical density of AT2 cells = N_AT2_/S.

### Clara cell numerical density

The Clara cells in the epithelium of terminal bronchiole distributed in the complete BADJ and alveolar ducts. The test cross line segments was overlaid in the photos. The area covered with line segments includes 324 intersections in 865 μm × 649 μm frame. The area of epithelial lamina was tested according to the right superior quadrant. The number of intersections falling on the epithelial lamina in the BADJ was counted concerning the criteria of test points in the alveolar wall surface. The adopted Clara cells express CCSP with a DAPI+ nucleus attached on the basilar membrane. The cell shape in the photos is cubical or prismatical. The surface fraction of epithelial lamina (F) = the number of adopted intersections on the epithelial lamina/324. The surface area of epithelial lamina (Se) = the surface of frame (865 μm × 649 μm) × F. The numerical density of Clara cells = number of adopted Clara cells (N_Clara_)/Se.

### Proliferating and apoptotic cell numerical density

Similar to the stereological analysis of AT2 cells, the Ki67^+^ proliferating cells and TUNEL^+^ apoptotic cells were counted respectively. The positive signals of adopted target cells reside in the nucleus. The numerical density of proliferating cells = the number of adopted Ki67^+^ cells (N_Ki67_)/S. The numerical density of apoptotic cells = the number of adopted TUNEL^+^ cells (N_TUNEL_)/S.

### Analysis of distribution characteristics in immunostaining cells

To further investigate the distribution regularity of positive granules (AQP5, Pro-SPC, CCSP, Ki67 and TUNEL), the three-dimensional structures were reconstructed under the guidance of the Image J software. Their distribution characteristics were intuitively presented using interactive 3D surface plots.

### Statistical analyses

All statistical calculations were conducted with SPSS 13.0 statistical programs. Quantitative data are given as mean ± SD. For statistical comparison, *one-way* ANOVA and *post-hoc* multiple comparisons and Chi-Square tests were used. Values of *P* < 0.05 were considered significant.

## Results

### Effect of RA-SS treatment on the general structure of lung tissues after ARDS

**Fig. 2:**
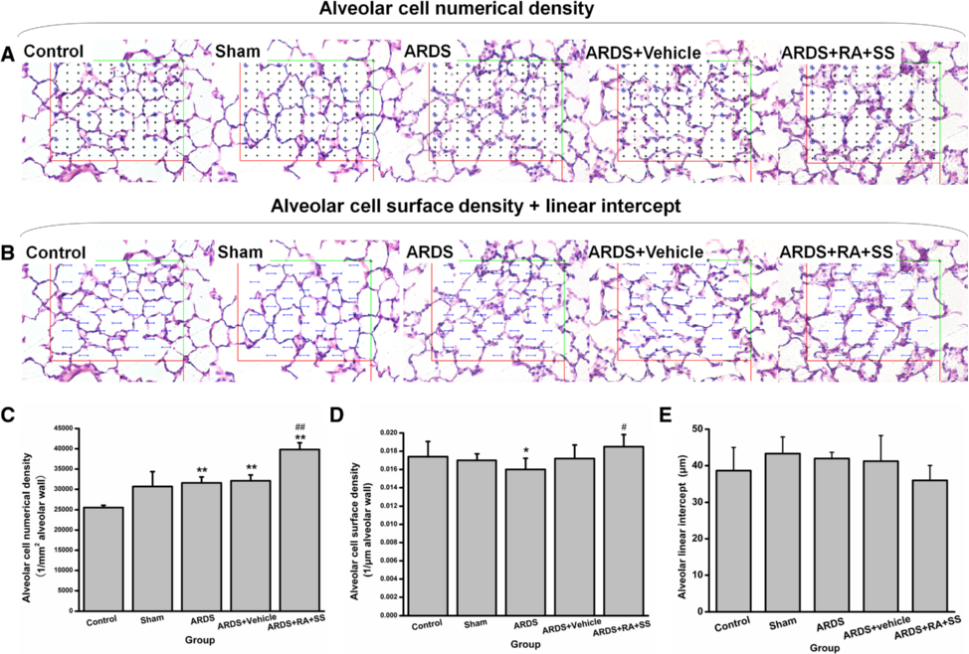
Effect of retinoic acid (RA) and simvastatin (SS) compound on the numerical density and surface density of alveolar cells and alveolar liner intercept (Lm) for acute respiratory distress syndrome (ARDS) in rats. **a** representative photos for analysis of alveolar cell numerical density. **b** representative photos for analysis of alveolar cell surface density and Lm. **c**, **d** and **e** statistical results for the numerical density and surface density of alveolar cells and alveolar Lm. * P < 0.05, ** P < 0.01 vs. the control group; ^*#*^ *P* < 0.05, ^*# #*^ *P* < 0.01 vs. the ARDS group

Pathological changes including the inflammatory infiltration and cell proliferation were found in the trachea and lungs in the ARDS group. The injured areas showed the focal distribution around the bronchioles, with the most significant in the right lower lobes. Treatment of RA-SS significantly attenuated the inflammatory infiltration and the proliferating changes in the pulmonary stroma. The number of alveolar cells was increased obviously in the ARDS + RA + SS group compared with the ARDS group. The general structural characteristics are near to those of the sham group, strongly suggesting that RA-SS compound could improve the inflammatory microenvironment as well as enhancing the alveolar repair in ARDS (Fig. 2a and b).

### Effect of RA-SS treatment on the lung tissues after ARDS by stereology

First, the numerical density of alveolar cells was decreased in the ARDS group than the sham and control groups (P < 0.05). RA-SS compound given intragastrically significantly reversed the reduction of numerical density of alveolar cells (P < 0.05) (Fig. 2c), indicating that RA-SS could increase the number of AT1 cells in ARDS. Second, the surface density of alveolar cells area was also decreased in the ARDS group compared with the sham and control groups (P < 0.05), which was reversed in the ARDS + RA + SS group (P < 0.05) (Fig. 2d). Meanwhile, Lm in the ARDS + RA + SS group was similar to that of other three groups (Fig. 2e). All of them indicated that RA-SS compound may enhance the repairing potential of alveolar cells while alveolar ectasia wasn’t found.

### Effect of RA-SS treatment on AT1 cells after ARDS by stereology

**Fig. 3:**
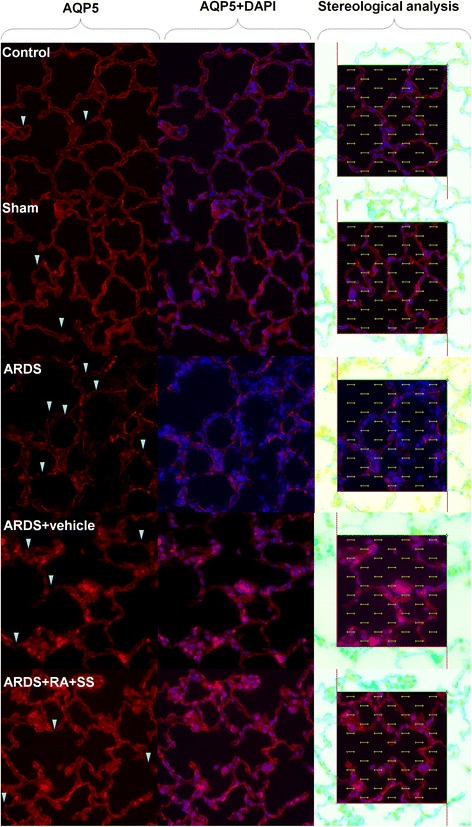
Effect of retinoic acid (RA) and simvastatin (SS) compound on the expression of AQP5 in alveolar cell type 1 (AT1) cells for acute respiratory distress syndrome (ARDS) in rats

**Fig. 4:**
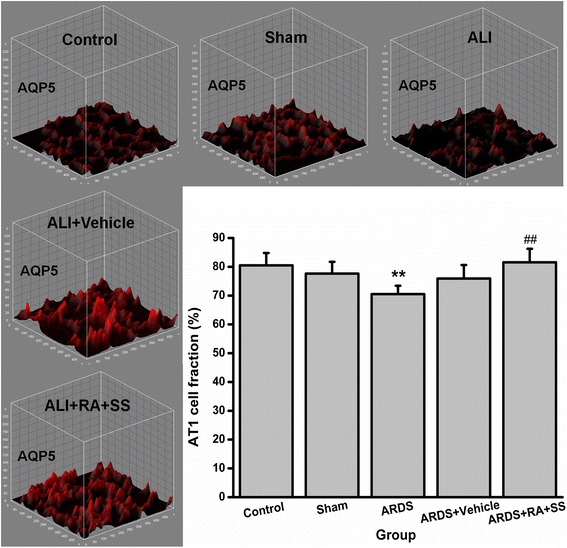
Effect of retinoic acid (RA) and simvastatin (SS) compound on the surface fraction and distribution characteristics of AQP5 in alveolar cell type 1 (AT1) cells for acute respiratory distress syndrome (ARDS) in rats. ** P < 0.01 vs. the control group; ^*#*^^*#*^ *P* < 0.01 vs. the ARDS group

The surface fraction of ATI cells was decreased in the ARDS group than the sham and control groups. However, it was obviously increased in the ARDS + RA + SS group than the ARDS group (Figs. 3 and 4), indicating that the administration of RA-SS compound may significantly promote the recovery of integrity in AT1 cells.

### Effect of RA-SS treatment on AT2 cells after ARDS by stereology

**Fig. 5:**
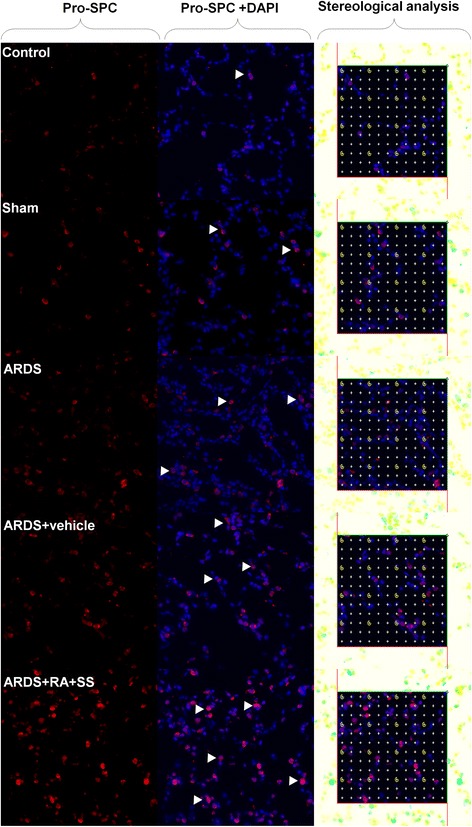
Effect of retinoic acid (RA) and simvastatin (SS) compound on the expression of Pro-SPC in alveolar cell type 2 (AT2) cells for acute respiratory distress syndrome (ARDS) in rats

**Fig. 6:**
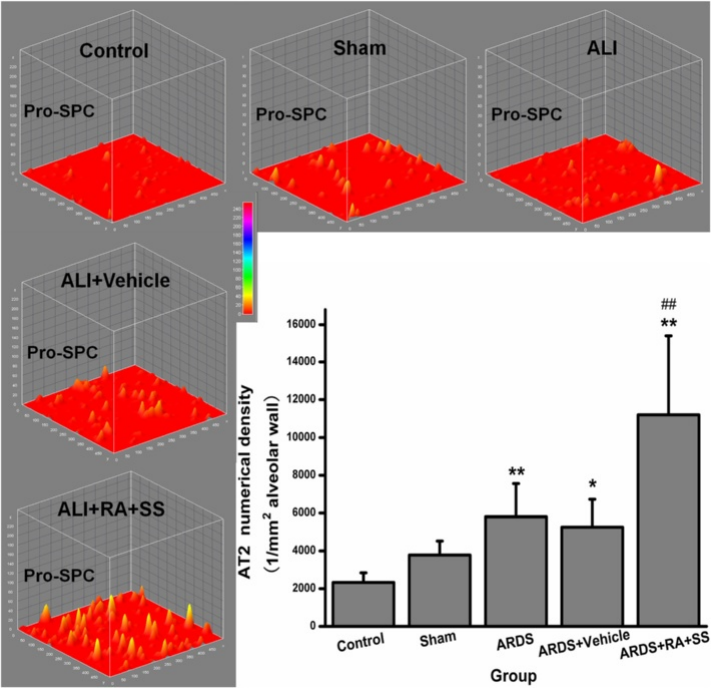
Effect of retinoic acid (RA) and simvastatin (SS) compound on the numerical density and distribution characteristics of Pro-SPC in alveolar cell type 1 (AT1) cells for acute respiratory distress syndrome (ARDS) in rats. * P < 0.05, ** P < 0.01 vs. the control group; ^*# #*^ *P* < 0.01 vs. the ARDS group

The numerical density of AT2 cells was increased in the ARDS group compared with the sham and control groups (P < 0.05). Moreover, it was further increased in the ARDS + RA + SS group (P < 0.05) (Figs. 5 and 6), suggesting that the RA-SS compound further enhance the repairing capacity in AT2 cells in ARDS.

### Effect of RA-SS treatment on Clara cells after ARDS by stereology

**Fig. 7:**
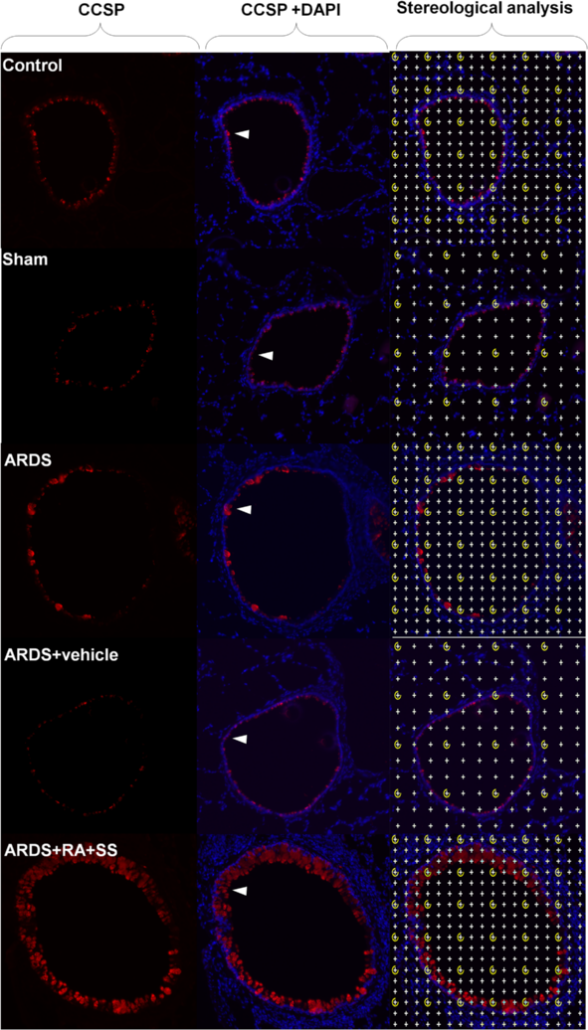
Effect of retinoic acid (RA) and simvastatin (SS) compound on the expression of CCSP in Clara cells for acute respiratory distress syndrome in rats

**Fig. 8:**
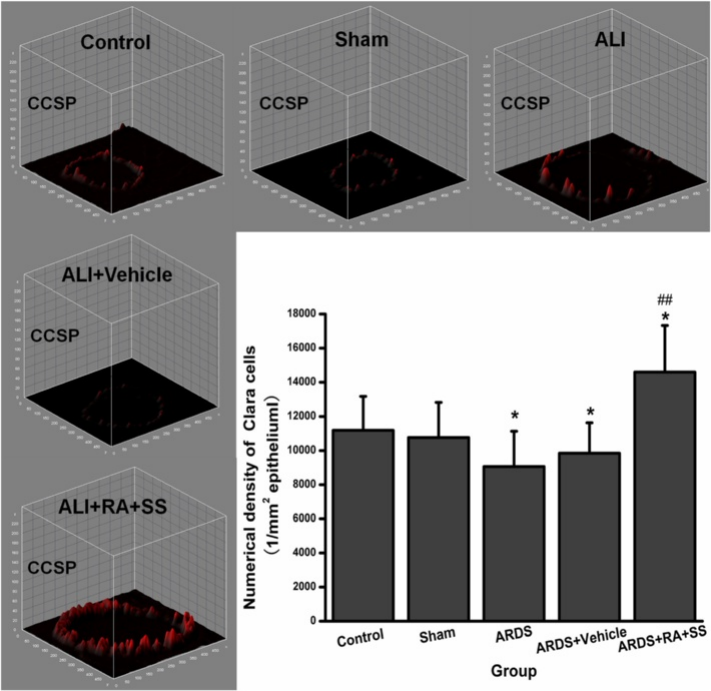
Effect of retinoic acid (RA) and simvastatin (SS) compound on the numerical density and distribution characteristics of CCSP in Clara cells for acute respiratory distress syndrome (ARDS) in rats. * P < 0.05 vs. the control group; ^*# #*^ *P* < 0.01 vs. the ARDS group

Meanwhile, the numerical density of Clara cells in the respiratory tract was decreased in the ARDS group than in the sham and control groups (P < 0.05), which was significantly reversed after the treatment of RA-SS compound. The numerical density of Clara cells was more in the ARDS + RA + SS group than the ARDS vehicle and ARDS groups (P < 0.05) (Figs. 7 and 8), indicating the promoting effect of RA-SS on the proliferation in Clara cells.

### Relationship between proliferation and apoptosis in breathing area after ARDS

**Fig. 9:**
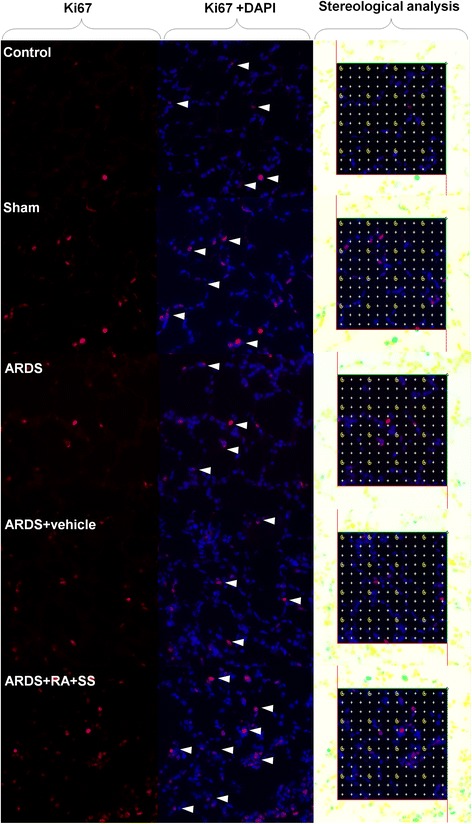
Effect of retinoic acid (RA) and simvastatin (SS) compound on the expression of Ki67 in alveolar cells for acute respiratory distress syndrome in rats

**Fig. 10:**
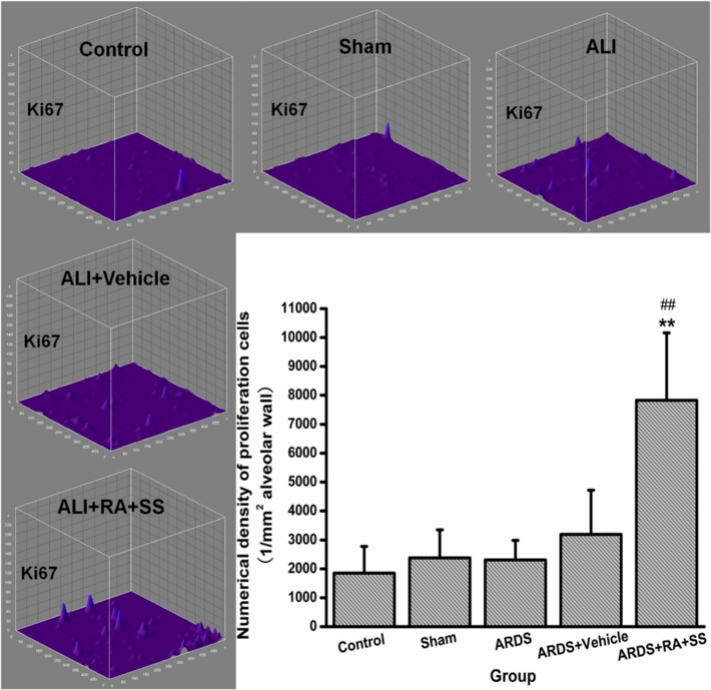
Effect of retinoic acid (RA) and simvastatin (SS) compound on the numerical density and distribution characteristics of Ki67 in alveolar cells for acute respiratory distress syndrome (ARDS) in rats. ** P < 0.01 vs. the control group; ^*# #*^ *P* < 0.01 vs. the ARDS group

**Fig. 11:**
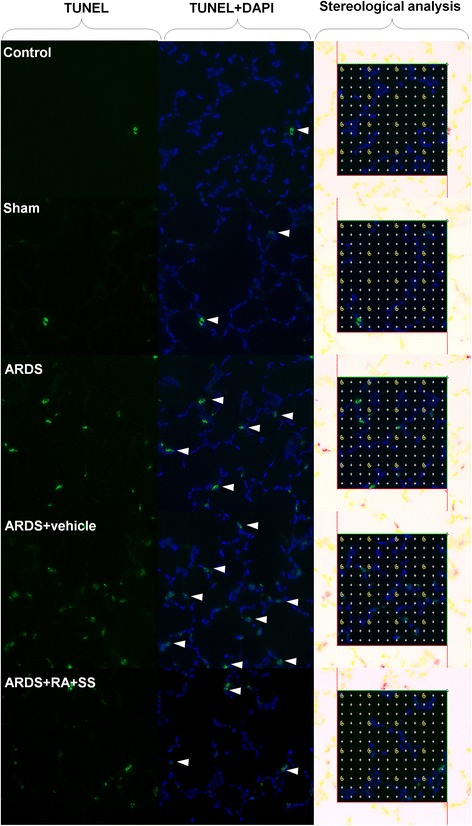
Effect of retinoic acid (RA) and simvastatin (SS) compound on the apoptosis in alveolar cells for acute respiratory distress syndrome in rats

**Fig. 12:**
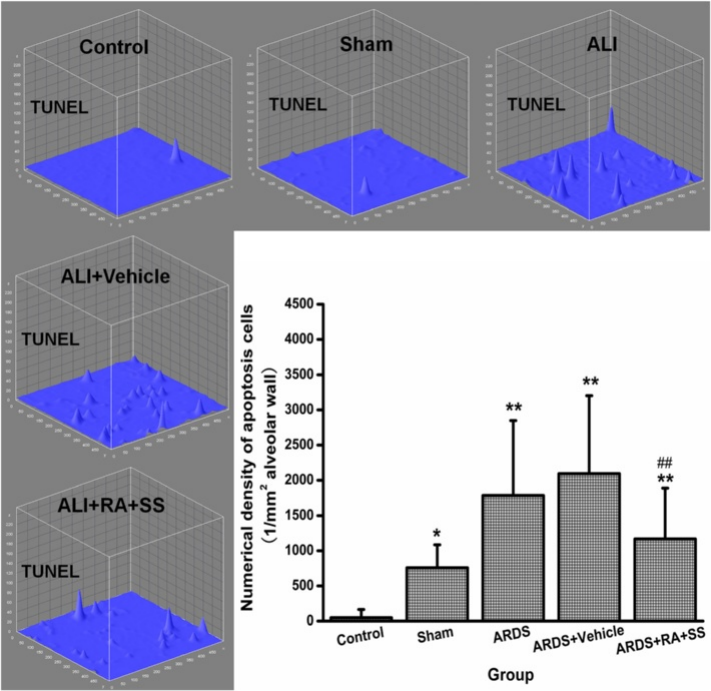
Effect of retinoic acid (RA) and simvastatin (SS) compound on the numerical density of apoptotic cells and distribution characteristics of TNUEL signals in alveolar walls for acute respiratory distress syndrome (ARDS) in rats. * P < 0.05, ** P < 0.01 vs. the control group; ^*# #*^ *P* < 0.01 vs. the ARDS group

To further elucidate the cellular mechanisms of the previous results, we examined the proliferation of alveolar cells as well as their late apoptosis. First, the numerical density of Ki67+ cells in alveolus showed no difference between the ARDS and sham or control groups, indicating the limited self repair capacity in ARDS. However, treatment of RA + SS significantly enhanced the numerical density of Ki67+ cells (P < 0.05) (Figs. 9 and 10). Second, the numerical density of TUNEL+ cells was increased in the ARDS group than the control and sham groups, which was efficiently inhibited in the ARDS + RA + SS group (P < 0.05) (Figs. 11 and 12), demonstrating its robust anti-apoptosis effect on the alveolar cells in ARDS. Concurrently, there exhibit an intrinsic relation between pro-proliferation and anti-late apoptosis courses, indicating its re-balancing role in the remodeling of injured lung tissues.

## Discussion

The present study used a rat model of hemorrhagic shock/resuscitation and intratracheal LPS injection to explore the role of RA-SS compound in the remodeling and regeneration of pulmonary tissues after ARDS. We demonstrated that RA-SS compound exerted anti-inflammatory and pro-repairing effects on respiratory tracts in ARDS. Our results also addressed the importance of the RA-SS compound - mediated re-balance between proliferation and late apoptosis in injured alveolar cells, which may clearly explain the dynamics of lung stem/progenitor cells in the lung repair.

Given the unsatisfied therapeutic efficacy of ARDS, the lung stem/progenitor cell-based endogenous repair has paved a new path on the repair/regeneration in the injured organs and tissues [7, 10, 27]. The efficient initiation of endogenous repair responses should include the protection of epithelial progenitors from injury and/or stimulation of endogenous progenitor cell function [7]. Thus, we selected RA, a pro-repair element, as well as SS, an anti-inflammatory and anti-apoptotic drug, to observe their synergistic role in the dynamic changes in the lung stem/progenitor cells and potential cellular mechanism. Results showed that the present doses of RA and SS had no obvious effect on the body weight of animals, indicating their biosafety within the experimental investigation.

Surprisingly, RA-SS compound could significantly increase the numerical density and surface density of alveolar cells, indicating its pro-repair efficacy in the alveolar remodeling in ARDS. Also, the elevated cell surface fraction of AT1 cells suggested the enhancement of cellular integrity and clearing capacity of edema fluid by AQP5 in alveolar sacs. Concurrently, the numerical density of AT2 cells increased in the ARDS group than the sham and control groups was further boosted after the RA-SS treatment, demonstrating the further enhancement of repair potential in AT2. Actually, The alveolar epithelium is composed of the flat type I cells comprising 95 % of the gas-exchange surface area and less than 5 % of cuboidal type II cells comprising the rest. New AT1 cells derived from rare, long-lived, mature AT2 cells that produce slowly expanding clonal foci of alveolar renewal. As a magic lung stem/progenitor cell population, AT2 cells possess randomly self-renewing and trans-differentiating capacity, which is broadly activated by acute AT1 injury via EGF signaling [10]. The increased AT2 cells not only contribute to the maintaining of alveolar integrity via covering the denuded area in ARDS, but also lower surface tension and prevent alveolar collapse via the boosted secretion of Pro-SPC, which was further confirmed by its distribution characteristics. In comparison, the contribution of AT2 cells is more significant than bronchioalveolar stem cells (BASCs) concerning the numerical preponderance and differentiation potential [28, 29]. Some murine AT2 cells can also generate BASCs unexpectedly [30]. Hence, although the numerical density of AT2 was increased in ARDS, it is of great necessity to be further boosted via the treatment of RA-SS compound.

Meanwhile, the decreased numerical density of Clara cells in ARDS was significantly reversed after the RA-SS treatment. As a critical airway progenitor cells, Clara cells possess anti-inflammatory capacity and impacts the pulmonary innate immune response [31, 32]. Conditional depletion of Clara cells induced peribronchiolar fibrosis, and potentiated lung inflammation and alveolar dysfunction, demonstrating its role of functional repair/regeneration in ARDS [33]. Concurrently, the analysis of CCSP distribution characteristics in Fig. 8 further showed that the boosted expression resulted in the transformation of cellular shape from sporadic cube to serried high prismatical, indicating the anti-inflammatory effect of CCSP was strengthened after RA-SS treatment. CCSP is an important lung derived protective factor and may play a substantial role on the pathogenesis of ARDS induced by endotoxemia [34]. Moreover, compelling evidence showed the differentiation potential of Clara cells towards AT1 and AT2 cells after severe lung injury [35, 36]. So, the improved AT1 and AT2 cells by stereological analysis maybe partly derived from facultative Clara cells so as to further keep the integrity of alveolar walls.

Given the role of RA-SS compound in the dynamics of AT1, AT2 and Clara cells, we further investigated the proliferating and apoptotic capacity of alveolar cells. Results showed that the numerical density of proliferating cells was significantly increased while that of apoptotic cells was decreased, clearly indicating that the imbalance of regeneration/apoptosis was at least partly recovered after RA-SS. Actually, the synergistic effect of RA and SS has been presented in the treatment other diseases [37, 38]. RA might play survival-promoting and apoptosis-inducing effects depending on the tissue types, doses, alternate activation of nuclear receptors and disease spectrum. RA might promote the proliferation of alveolar cells via highly expressed nuclear receptor, fatty acid binding protein 5 (FABP5) [39], as well as the downregulation of Cdk inhibitory protein, p21 [40], while SS could reverse the inflammatory injury and apoptosis, enhancing the restoration of alveolar cell function via the HMG-CoA reductase-dependent activation of PI3K/Akt signaling, reduction of neutrophil recruitment and radical form [41–43]. Recently, large clinical trials of statins for sepsis associated ARDS have resulted in negative results. We viewed that the results of animal and clinical studies are occasionally different owing to the differential metabolism system and biological responses. Also, in these studies, the negative outcome might be, at least in part, due to the concurrent or recent use of systemic glucocorticoid therapy. The synergistic treatment of RA and SS may be of great necessity. Hence, the prophylactic and therapeutic effects of RA-SS in ARDS are possibly related to the amelioration of shock-endotoxin-induced pulmonary damage and inhibition of apoptosis as well as enhancement of functional repair/regeneration.

## Conclusions

**Fig. 13:**
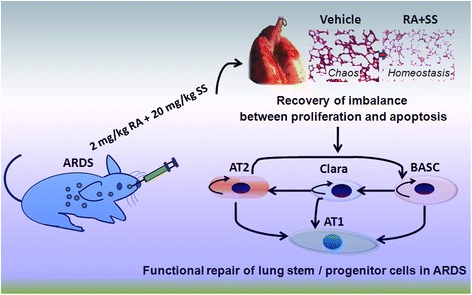
Schematic view of the role of retinoic acid (RA) and simvastatin (SS) compound in the functional repair of lung stem/progenitor cells in the present study. RA and SS are synergistically involved the remodeling of injured pulmonary tissues. The possible mechanisms refer to the re-balance between regeneration and apoptosis in lung stem/progenitor cells, which is helpful for the pulmonary remodeling/regeneration

Taken together, treatment of RA plus SS had obvious beneficial effect on the remodeling of injured pulmonary tissues, suggesting that the underlying mechanisms are related to the re-balance between regeneration and apoptosis in lung stem/progenitor cells (Fig. 13). Additionally, it is valuable to further elucidate the mechanisms of action of RA on proliferation/anti-apoptotic effects post ARDS with RARa mice. Future studies should also focus on the detailed repair mechanism via in vivo lineage tracing technique and ex vivo clonal analysis in pulmonary stem/progenitor cells in the treatment of RA-SS.

## Abbreviations

ARDS: Acute respiratory distress syndrome
ATRA: All-trans retinoic acid
RA: Retinoic acid
AT1: Alveolar type 1
AT2: Alveolar type 1
BP: Blood pressure
FABP5: Fatty acid binding protein 5
HE: hematoxylin and eosin
HR: Hemorrhagic shock/resuscitation
HMG-CoA: 3-hydroxy-3-methylglutaryl coenzyme A
Lm: Liner intercept
PBS: Phosphate-buffered saline
SS: Simvastatin
TUNEL: Terminal deoxylnucleotidyl transferase mediated-dUTP nick end labeling
UIR: Uniform isotropic random
